# Exploring the barriers to mental health service utilization in the Bolgatanga Municipality: the perspectives of family caregivers, service providers, and mental health administrators

**DOI:** 10.1186/s12913-024-10567-2

**Published:** 2024-03-05

**Authors:** Dennis Bomansang Daliri, Gifty Apiung Aninanya, Timothy Tienbia Laari, Nancy Abagye, Richard Dei-Asamoa, Agani Afaya

**Affiliations:** 1 Presbyterian Psychiatric Hospital, Bolgatanga, Ghana; 2 Department of Global and International Health, School of Public Health, University for Development Studies, Tamale, Ghana; 3 Department of Health Services Policy Planning Management and Economics, School of Public Health, University for Development Studies, Tamale, Ghana; 4 Presbyterian Primary Health Care, Bolgatanga, Ghana; 5 Department of Midwifery, School of Nursing and Midwifery, University of Ghana, Legon, Ghana; 6 Department of Psychiatry, Korle-Bu Teaching Hospital, Accra, Ghana; 7 Department of Psychiatry, University of Ghana Medical School, Accra, Ghana; 8 Department of Nursing, School of Nursing and Midwifery, University of Health and Allied Sciences, Ho, Ghana

**Keywords:** Mental health service utilization, Family caregivers, Financial challenges, Service providers

## Abstract

**Background:**

Mental health service utilization remains a challenge in developing countries, with numerous barriers affecting access to care. Albeit data suggest poor utilization of mental health services in the Bolgatanga Municipality in Ghana, no studies have explored the barriers to the utilization of mental health services. Therefore, this study explored the perspectives of family caregivers, service providers, and mental health service administrators on the barriers to mental health service utilization in the Bolgatanga Municipality, Ghana.

**Methods:**

A qualitative descriptive design was employed for the study. Nineteen participants were purposively sampled from two hospitals including fifteen family caregivers, two service providers, and two mental health administrators. Data were collected through individual in-depth interviews using a semi-structured interview guide. Audio-recorded interviews were transcribed verbatim and thematically analyzed using NVivo 12 pro software.

**Results:**

Five main themes emerged including individual-level barriers, interpersonal barriers, community-level barriers, organizational-level barriers, and policy-level barriers. At the individual level, lack of insight, poor treatment compliance, and financial challenges were prominent barriers. Interpersonal barriers included family pressure influenced by cultural and spiritual beliefs. At the community level, stigma and mental health illiteracy were identified as significant barriers. At the organizational level, barriers encompassed inadequate staffing, limited space, and staff attitudes. Policy-level barriers included the neglect of mental health in policies and the non-inclusion of mental health services in the National Health Insurance Scheme.

**Conclusion:**

This study highlights the complexity of barriers to mental health service utilization and underscores the need for a comprehensive approach to address them. Collaborative efforts involving healthcare providers, policymakers, communities, and families are essential to mitigate these barriers. It is imperative to consider these barriers when developing strategies to improve the utilization of mental health services in Ghana.

**Supplementary Information:**

The online version contains supplementary material available at 10.1186/s12913-024-10567-2.

## Introduction

It is estimated that approximately 85% of people with mental health conditions in developing countries do not utilize mental health services (MHS) [1]. This implies that the majority of people with mental health conditions living in developing countries such as Ghana do not receive appropriate evidence-based mental healthcare. In Nigeria for instance, only about 10.4% of persons with mental health conditions received care from MHS [2]. Considering that an estimated 80% of persons with mental illness live in low-and-middle-income countries, such a poor statistic on utilization of MHS poses a serious global health challenge [3].

Mental health conditions are also projected to increase exponentially by 2030 [4]. The main drivers of this exponential increase are poverty, migration, conflict, and lifestyle changes such as substance abuse, sedentary lifestyle, poor diet, and chronic stress, all of which are endemic issues in low- and middle-income countries. To resolve the potential challenges that these will pose to mental healthcare, steps must be taken to ensure increased availability and utilization of MHS. This requires an in-depth understanding of current barriers to the utilization of MHS by persons with mental health conditions, especially in the global south.

Studies conducted in various parts of Africa and the world show diverse barriers to the utilization of MHS. For instance, a study in Nepal identified a lack of awareness of available services and stigma as major barriers to MHS utilization in the country [5]. Also, a recent study in Kenya reiterated the role of stigma as a barrier to accessing MHS [6]. The study further showed that over 50% of participants who attend outpatient clinics miss their visits largely because of financial challenges, demonstrating that the cost of accessing care also serves as a barrier to the utilization of MHS especially in African countries. This is further buttressed by statistics from the World Health Organization’s 2020 Mental Health Atlas which show that in 41% of African countries, patients who seek mental healthcare services pay entirely out of pocket [7].

In a similar study in Sudan, the cost of services and availability of service providers were again identified as barriers. However, major barriers that were identified included beliefs regarding mental health disorders and the use of spiritual and traditional healing [8]. Within the African context, cultural and religious beliefs significantly impact the health-seeking behavior of populations and tend to affect the utilization of MHS [9, 10]. Mental disorders are often attributed to spiritual causes such as curses, witchcraft, “Satan’s work”, or punishment from God [11–13]. These beliefs are not confined to uneducated or rural dwellers as such beliefs have been noted among educated populations, including health workers [12].

A study conducted in the Niger Delta Region of Nigeria involving service users further corroborates the role of cultural beliefs, cost, and availability of services as well as stigma as barriers to accessing MHS [14]. Another barrier to the utilization of mental health services as reported by patients in Rwanda was geographical inaccessibility [15]. This is further corroborated by Devkota et al. [5] who reported that caregivers in Nepal found the distance from their communities to the mental health service as a challenge which impeded their service utilization.

Considering the diverse barriers identified in the above studies, it is essential to identify context-specific barriers as these may vary across settings. Also, to better understand these barriers, the views, and experiences of stakeholders such as caregivers are invaluable. This is particularly important since in African settings caregivers tend to largely contribute to determining whether or not patients access MHS [16].

Another set of key informants whose insights are relevant to identifying and removing barriers to MHS are service providers and mental health service administrators. These professionals tend to have extensive experience with these barriers and their effects on the patients they manage. Within the African context, where the availability of such service providers has been cited as a major barrier, their involvement in evaluating and removing these barriers has tremendous value [17].

Despite the above, few studies have evaluated the perspective of caregivers and service providers in Ghana regarding the barriers to the utilization of MHS within the communities in the country. Although unpublished evidence from the District Health Information Management System (DHIMS-2) suggests poor utilization of MHS in the Bolgatanga Municipality, no studies are currently available that assess the barriers that persons with mental illness face in the utilization of MHS. This study therefore aimed to fill this gap in knowledge by exploring the perspectives of family caregivers, service providers, and mental health administrators on barriers to MHS utilization within the municipality. Ultimately, it answers the research question “what are the barriers to MHS utilization from the perspective of family caregivers, service providers, and mental health administrators in the Bolgatanga municipality?”

## Methods

### Study design

We used qualitative descriptive study design to explore the barriers to mental health service utilization without subjecting the data to any interpretive depth [18]. The report of the study followed the 32-item checklist of the Consolidated Criteria for Reporting Qualitative Research (COREQ) [19].

### Study setting

According to WHO estimates, approximately 13% of Ghanaians suffer from mental disorders, with 3% suffering from a severe mental disorder and 10% from a moderate to mild disorder [20]. With a treatment gap of 98%, many people with mental illness do not get the appropriate care [20]. Till now, anecdotal evidence shows that Ghana can only boast of a little over 60 psychiatrists for a population of thirty-one million [21]. Most of these psychiatrists are in the bigger cities such as Accra and Kumasi. Attempts have been made to improve mental healthcare with the passage of the Mental Health Act (Act 846) which seeks to enhance community care by encouraging the integration of mental health care into primary health care shifting away from the traditional institutionalization [22]. Mental health in Ghana still faces challenges including inadequate funding, non-inclusion onto the National Health Insurance Scheme [NHIS], inadequate resources among others [23].

The study was conducted in the Bolgatanga Municipality, Ghana. The municipality has a population of 139,864 (73,257 females and 66,607 males) with nearly 64% of the total population living in urban areas [24]. The inhabitants of the municipality seek MHS mainly from the Presbyterian Psychiatric Hospital and the Upper East Regional Hospital. These two secondary-level facilities in the municipality were purposively chosen for this study because they are the only facilities that provide MHS. The Presbyterian Psychiatric Hospital in the Bolgatanga Municipality is the only psychiatric hospital in the northern/savannah ecological zone of Ghana. The hospital is staffed with one psychiatrist, mental health nurses, and support staff. The hospital provides out-patient, in-patient, and community mental health services to patients with mental illness and their families. Being the only psychiatric hospital in the region, the hospital serves as the major referral point for the five regions of northern Ghana and parts of some neighboring countries like Burkina Faso and the Republic of Togo. The Upper East Regional Hospital also has a mental health department that renders mental health care to patients with mental illness, mostly by mental health nurses.

### Population and inclusion criteria

Family caregivers of people with mental illness, service providers, and mental health administrators were the target population. Family caregivers who were 18 years or older, had at least six months of caregiving experience, and consented to participate were included in the study. Mental health Service providers and administrators with at least six months working experience were included. However, those who were MHS users themselves were excluded from the study. This exclusion was to achieve a homogeneous group to reduce confounders that could complicate data interpretation.

### Sampling method and sample size

A purposive sampling method was employed to select participants based on their experiences with MHS [25]. Potential participants who met the inclusion criteria were contacted face-to-face by the lead researcher to discuss the purpose of the study. Those who voluntarily consented were included in the study. The Presbyterian Psychiatric Hospital sees most of the patients with mental illness followed by the Upper East Regional Hospital. Nine family caregivers were interviewed at the psychiatric hospital and six were interviewed at the Upper East Regional Hospital. The study included one nurse from each of the hospitals, the municipal mental health coordinator, and the regional mental health coordinator. In all, a total of nineteen participants were interviewed for the study. Data saturation, the point where no new information emerged determined the sample size [26] and no participant dropped out of the study.

### Data collection tool and method

A semi-structured interview guide adopted from a previous study in Nepal [5] and adapted to suit the study setting (since the adopted study was carried out in primary healthcare facilities) was used to collect data for the current study. Prior to data collection, the interview guide was piloted with three participants (one caregiver, one service provider, and one mental health administrator) to identify and correct mistakes. From February to March 2023, the lead researcher, a male medical doctor with experience in psychiatry and qualitative studies conducted individual in-depth interviews in secluded offices in the hospitals during less busy periods (determined by participants). The interviewer had no prior relationship with the participants. A total of nineteen interviews were conducted lasting 30 to 45 minutes. Fifteen interviews were in the English language and four were in Grune (the predominant local language). These two languages were used based on the participants’ preferences. Open-ended questions were asked during the interviews and probes were used to elicit responses. Adequate time was allowed for the participants to expansively express their views on the barriers to MHS utilization. Iterative questioning was also employed to clarify unclear responses. Field notes of non-verbal expressions were taken, the interviews were audio recorded, and no repeat interviews were conducted.

### Data analysis

The data were analyzed through thematic analysis using NVivo 12 pro software. Verbatim transcription of the recorded interviews was done immediately after each interview. Following the approach of Braun and Clarke [27] the transcripts were read several times by the first and second authors to familiarize themselves with the data. The transcribed data were imported onto the NVivo 12 pro software with line-by-line reading and coding done. The first and second authors discussed the codes and disagreements were resolved. Phrases about the barriers to MHS utilization relevant to the codes were identified and collated. Probable themes were searched for and the relationships between the codes and themes were identified to form the main themes. Some themes were modified, and others collapsed into sub-themes. The candidate themes were then defined, further refined, and the data within them were analyzed. The report was then written from the data to present the narratives in a clear and logical manner within the themes. For easy retrieval of data, soft copies of anonymized transcripts were saved in a password-protected computer.

### Rigor

To ensure rigor, Lincoln and Guba’s framework of credibility, dependability, confirmability, and transferability was applied [28]. To ensure confirmability, the transcripts were returned to the participants for validation (member checking) [29]. To ensure transferability, sufficient details about the study’s setting, sample, and processes were provided (thick description) [30, 31]. To ensure credibility, data were collected from multiple sites (space triangulation) [32] and different persons (person triangulation) [33]. To ensure dependability, peer debriefing and strict adherence to verbatim transcriptions were done [34].

### Ethical considerations

The study was approved by the Committee on Human Research, Publication and Ethics (CHRPE) of the Kwame Nkrumah University of Science and Technology (reference number: CHRE/AP/038/23). Also, written institutional permission was granted by the Upper East Regional Hospital and the Presbyterian Psychiatric Hospital. The participants were told about their right to voluntarily participate and withdraw without penalties. All the participants gave written consent and signed the informed consent form before the interviews. The privacy, confidentiality, and anonymity of participants were protected throughout the study by assigning P1…P15 to Family caregivers and KI1…KI4 to service providers and MHS administrators. This study was conducted in line with the Declaration of Helsinki ethical principles for medical research involving human subjects.

## Results

### Demographic characteristics of participants

Participants in this study were made up of 15 family caregivers of patients with mental illness, two service providers, and two MHS administrators in the Bolgatanga Municipality. Participants were mainly females with ages ranging from 20 to 68 as shown in Table 1.

**Table 1:** Characteristics of participants

Participants (P) / Key Informant (KI)	Age (Years)	Sex	Educational level	Occupation	Relationship With Patient
P1	45	Female	Tertiary	Teacher	Mother
P2	68	Female	Tertiary	Retired teacher	Mother
P3	40	Male	Tertiary	Civil servant	Husband
P4	38	Female	Uneducated	Unemployed	Mother
P5	20	Male	Secondary School	Apprentice	Nephew
P6	58	Female	Junior High school	Trader	Mother
P7	26	Female	Secondary School	Apprentice	Wife
P8	57	Male	Tertiary	Pastor	Husband
P9	43	Female	Primary	Trader	Mother
P10	59	Female	Junior High School	Seamstress	Mother
P11	33	Male	Tertiary	Entrepreneur	Brother
P12	27	Male	Secondary School	Mason	Brother
P13	56	Female	Uneducated	Trader	Mother
P14	32	Male	Tertiary	Nurse	Brother
P15	39	Male	Junior High School	Security Officer	Brother
KI1	35	Female	Tertiary	Nurse	Service Provider
KI2	33	Female	Tertiary	Nurse	Service Provider
KI3	40	Male	Tertiary	Nurse	Mental Health Administrator
KI4	43	Male	Tertiary	Nurse	Mental Health Administrator

### Barriers to the utilization of mental health services

Five main themes emerged from the analysis of the data including individual-level barriers, interpersonal barriers, community-level barriers, organizational-level barriers, and policy-level barriers. The themes with their corresponding sub-themes are in Table 2.

**Table 2:** Themes and sub-themes

Main themes	Sub-themes
Individual-level barriers	1. Lack of insight 2. Financial challenge 3. Poor compliance with treatment 4. Beliefs about the causes of mental illness
Interpersonal barriers	1. Family pressure
Community-level barriers	1. Stigma 2. Mental health illiteracy
Organizational-level barriers	1. Inadequate staffing and space 2. Staff attitude
Policy-level barriers	1. Neglect of mental health 2. Non-inclusion of mental health services on to the NHIS

Abbreviation: NHIS- National Health Insurance Scheme

### Individual-level barriers

The study revealed some barriers at the individual level that hindered the utilization of MHS in the Bolgatanga Municipality. While some participants attributed the poor utilization of MHS to the lack of insight, others attributed it to financial challenges, poor treatment compliance, and beliefs about the causes of mental illness. 

#### Lack of insight

The participants mentioned that a barrier to the utilization of MHS was the lack of insight into the illness and this led to denial of the condition by the ill relative. This made it difficult to bring them for MHS as they claimed they were not ill.

*“First when they sent him to the hospital in Tamale and was discharged his mind was that he wasn’t sick. That if he was sick, they would have kept him there for a long time but because they admitted him for a short time, he said he wasn’t sick so wouldn’t come to the hospital” (P5, male caregiver).*

*“…Yeah, like he doesn’t want to come to the hospital, he says there is nothing wrong with him. I even told him that if it was about money, he shouldn’t worry because the family was ready to help him” (P7, female caregiver).*

#### Financial challenge

Almost all participants complained about the high cost of MHS being a disincentive in getting them to bring their relatives for the service..

*“There is nothing that discourages me except that this problem is financially draining and very expensive otherwise, there is no way I will not seek care for my son” (P1, female caregiver).*

*“Financial challenges have been my main problem, anytime we have to bring him, we will need to get a vehicle, pay for the transportation and also pay for the medications which have become a challenge for me” (P13, female caregiver).*

#### Poor compliance with treatment

Some participants mentioned that the poor compliance of their relatives to the treatment discouraged them from bringing them for the service.

*“He refuses to take his medicine, that is the problem. As for the other things about money, we don’t think that is a problem. If only he will take the medicine and get well, we will be very happy. This attitude sometimes makes me uninterested to bring him for care” (P12, male caregiver).*

#### Beliefs about the causes of mental illness

What participants believed to be the cause of the mental illness of their relatives influenced their utilization of MHS. Some participants reported that they did not use the service because they believed mental illness had a spiritual cause.

*“I first sent him to the church for prayers because I did not think it could be a medical condition but spiritual hence, I sent him to the church” (P4, female caregiver).*

*“She was first sent to the spiritual healer (fetish priest) in her village from Accra because the symptoms were unusual, we had never seen anything like this, and we heard that it was spiritual” (P13, female caregiver).*

### Interpersonal barriers

Interpersonal barriers to the utilization of MHS were also revealed in the study. The participants mentioned family pressure as their main interpersonal barrier to the utilization of MHS.

*“Another one was the influence from family where they even pushed me to leave him at a church for prayers for a period of one week even against my will” (P9, female caregiver)*.

*“My family members have been on me; they keep telling me that my son’s condition is spiritual so I should send him to a spiritualist and not a hospital” (P2, female caregiver).*

### Community-level barriers

Some community-level barriers to the utilization of MHS were revealed in the study. Stigma and mental health illiteracy were barriers at the community level that discouraged the utilization of MHS.

#### Stigma

The participants mentioned that stigma from the community was a major barrier to the utilization of MHS. A participant intimated that but for her resilience and unique nature, she would not have been able to withstand the stigma from her community and hence could not have sent her relative for care.

*“Another challenge is the stigma, because of the accusation of witchcraft and the beliefs about mental illness in my community, I sometimes feel like staying home and keeping my daughter from outsiders” (P6, female caregiver).*

*“I have been a unique parent; I didn’t care about stigma or anything. I always go with him to the hospital and have pushed away everything that people say” (P2, female caregiver).*

#### Mental health illiteracy

Most of the participants admitted that their communities lacked the requisite awareness of mental health, and this led to people keeping patients at home instead of bringing them for mental health care. They further mentioned that their ignorance about mental health was the reason why they resorted to traditional and faith-based healers.

*“I have been ignorant about mental health and even more not knowing there is a place for treatment such as this” (P14, male caregiver).*

In corroborating the above statement, a service provider stated that ignorance about mental health was the reason why people don’t use MHS and further opined that more education could solve it.

*“Education should be intensified since most of our people are ignorant about mental health hence resort to faith healers” (KI 1, female service provider).*

### Organizational-level barriers

Some organizational barriers to the utilization of MHS were revealed in the study. These barriers were mainly on the health facilities and the personnel working in the facilities. The participants mentioned inadequate staffing, lack of space, and staff attitude as organizational barriers in the utilization of MHS.

#### Inadequate staffing and space

Most of the family caregivers complained about inadequate staffing and space for admitting persons with mental illness, which discouraged them from bringing their relatives for admission and care at the mental health facilities.

A service provider mentioned that inadequate staffing of the mental health facilities is a challenge for the utilization of the service.

*“There is the need to step up the staffing levels to raise the level of services provided in the municipality since this leads to unnecessary delays at the facilities and clients complain bitterly” (KI 1, female service provider).*

A caregiver also narrated how inadequate space in the facilities hinders the utilization of MHS in the Municipality.

*“I think there is a need for expansion of the mental health service so that more people can be admitted if they need the service since being returned for lack of space discourages us” (P15, male caregiver).*

#### Staff attitude

Even though some participants agreed that the staff of the mental health facilities are very supportive and professional, others reported that there was a need for attitudinal change among some members of staff. One participant described the attitude of some mental health nurses as discouraging.

*“Also, the attitude of nurses is so discouraging; the way they look at us when we mention that we are coming to the psychiatric hospital is so appalling” (P1, male caregiver).*

Another caregiver reported that the nursing staff needed to be more professional.

*“I think the nursing staff should be given a little more training to be well abreast with these conditions. Brain conditions are very stressful hence mistakes need to be avoided” (P1, female caregiver).*

### Policy-level barriers

Some barriers at the policy level were reported to hinder the utilization of MHS. These were centered mainly on policies formulated at the national level. The participants mentioned the neglect of mental health and the non-inclusion of MHS onto the NHIS as barriers.

#### Neglect of mental health

A participant lamented about the lack of attention at the policy level to support MHS in the country and this is a barrier to the utilization of the service.

*“The sector has been neglected somehow; government doesn’t pay so much attention to the condition. even the district assembly common fund, mental health has a percentage, but they rather concentrate on LEAP (livelihood empowerment program) and HIV (Human immune-deficiency virus) instead of considering mental health, and that is very bad because it is a critical sector” (P3, male caregiver).*

#### Non-inclusion of mental health services on to the national health insurance scheme

All the participants lamented the lack of health insurance coverage for mental illnesses. This, they said has made the utilization of MHS very expensive and hence a barrier to the use of the service.

*“…if the government could absorb the medication cost or cover the service with a health insurance package, it would encourage people to keep coming” (KI 1, female service provider).*

*“Also, the non-inclusion of mental health into the health insurance scheme. These people are the vulnerable in society, but they have been excluded” (MH Administrator 2).*

## Discussion

The study explored the barriers to the utilization of MHS in the Bolgatanga Municipality, Ghana. The findings from the study shed light on the intricate web of barriers to the effective utilization of MHS. The results highlight several barriers across individual, interpersonal, community, organization, and policy levels collectively shaping the landscape of mental health care access.

Lack of insight into the illness, poor treatment compliance, and financial challenges emerged as barriers at the individual level that discouraged the utilization of MHS. This finding is supported by an earlier scoping review which identified attitudinal barriers (poor compliance and poor insight), and structural barriers (cost of service) as barriers to the utilization of MHS [35]. The denial of illness by affected individuals presents a significant hurdle, as caregivers struggle to facilitate treatment when the individual does not perceive the need for it [5]. This emphasizes the importance of raising awareness and education about mental health within families to overcome this lack of insight [36]. Furthermore, the financial burden associated with mental health care underscores the need for accessible and affordable services. Policy reforms that prioritize mental health within healthcare funding could alleviate this financial stress, encouraging more families to seek professional help.

Family pressure, often rooted in cultural or spiritual beliefs, influences caregiving decisions, sometimes diverting families from conventional medical care towards alternative remedies. Studies have confirmed that family members may express negative attitudes towards MHS thereby inhibiting its utilization [37]. Addressing this issue involves sensitizing families to the value of evidence-based mental health interventions. Education campaigns within communities can counter misinformation and encourage open dialogues regarding mental health, diminishing the impact of family pressures on caregiving choices. This has become very important since studies have shown that mental health information found on social media was erroneous [38, 39].

Stigma remains a significant community-level barrier to MHS utilization. Caregivers expressed concerns about societal perceptions and accusations, leading some to keep their relatives with mental illness hidden from public view. This finding has been reported by several studies [9, 16, 40]. Interestingly, self-stigma has been reported among caregivers as one of the hindrances to the utilization of MHS [5]. This, however, was not identified in this study. The issue of stigma indicates the necessity of implementing anti-stigma campaigns that not only destigmatize mental illnesses but also emphasize the importance of seeking professional help. Additionally, mental health illiteracy within communities encourages reliance on traditional or faith-based healers, underscoring the urgency of disseminating accurate mental health information to the public [41].

Inadequate staffing, space, and suboptimal staff attitudes within mental health facilities emerged as organizational-level barriers. This finding has been corroborated by studies from Pakistan [9] and Nepal [5] who reported similar findings among family caregivers. Insufficient resources hinder the seamless delivery of care, potentially leading to discouragement among family caregivers. Addressing these concerns necessitates infrastructure improvements and capacity building within mental health services [42]. Enhancing staff attitudes through training and sensitization could foster a more empathetic and accommodating environment, facilitating caregivers’ engagement with MHS [43].

The neglect of mental health within national policies and the exclusion of MHS from health insurance schemes pose substantial challenges. This finding has been affirmed by a Nigerian study which reported that the mental health sector has been neglected and is evidenced by the lack of legislation and policies to regulate the MHS [44]. Recognizing mental health as an integral aspect of overall well-being is critical for reform. Advocating for policy changes that increase funding and insurance coverage for mental health services could bridge existing gaps and promote more equitable access to care.

### Strengths and limitation of the study

The study’s findings shed light on critical barriers to MHS utilization in a specific municipality, which can inform policy development and interventions aimed at improving MHS utilization. By focusing on the Bolgatanga Municipality in Ghana, the study provides context-specific information, which is valuable for addressing local mental health issues and tailoring interventions to the specific needs of the population. Despite these strengths, the study has a limitation: participants may have provided responses that they believed were socially acceptable or desirable, potentially leading to underreporting of sensitive issues or overemphasis on certain barriers.

## Conclusion

This study underscores the multifaceted nature of barriers to MHS utilization. To enhance the engagement of family caregivers, individuals with mental illness, and communities with MHS, a holistic approach is required. Collaborative efforts involving healthcare providers, policymakers, communities, and families can work collectively to mitigate these demotivating factors. By addressing these barriers comprehensively, societies can foster a culture of mental health support and inclusivity, ultimately improving the overall well-being of individuals and families dealing with mental health conditions. We recommend further research to explore potential solutions and interventions to address these barriers effectively.

### Electronic supplementary material

Below is the link to the electronic supplementary material.

Supplementary Material 1

## Data Availability

Data for the study can be found in the study as excerpts. Full data will be available from the corresponding author upon reasonable request.

## Abbreviations

COREQ: Consolidated Criteria for Reporting Qualitative Research
DHIMS-2: District Health Information Management systems
MHS: Mental Health Services
NHIS: National Health Insurance Scheme
