# Supplementary material for: Exploring the barriers to mental health service utilization in the Bolgatanga Municipality: the perspectives of family caregivers, service providers, and mental health administrators
Source: BMC Health Serv Res. 2024 Mar 5;24:278. doi: 10.1186/s12913-024-10567-2 (PMC10916073; doi:10.1186/s12913-024-10567-2)
Supplement: Supplementary file 1 — Supplementary Material 1 [file 12913_2024_10567_MOESM1_ESM.docx]

**IN-DEPTH** **INTERVIEW GUIDES**

**Interview guide for Family Caregivers of mentally ill patients**

**Introduction**

I am conducting a study to assess the Family caregivers’ experiences with the utilization of mental health services in selected health facilities in the Bolgatanga municipality. I will be grateful if you could spend a little of your time completing this interview with me. There are no right or wrong answers. Any information provided is private and confidential. This study is only for academic purposes. Your participation in this study is entirely voluntary. Please feel free to answer the questions below. Thank you.

Participant Code……………. Date of interview………………

**Demographics**

**The following questions will be about your demographic characteristics (age, marital status, etc.).**

1. Can you please tell me your age?

2. What gender are you?

3. What is your ethnicity?

4. What is your marital status?

5. How many children do you have?

6. Are you educated? To What level please?

7. What work do you do?

8. What is your place of residence?

9. What is your religion?

10. How are you related to the patient?

| **Topics and Issues** | **Main Question** | **Follow up questions** | **Probe** |
| --- | --- | --- | --- |
| Ice-breaker  Perception of mental illness | Please introduce yourself | 1. Before your relative got mentally ill, what was your thinking regarding mental illness? 2. What about your relative made you think he/she was mentally ill? 3. How do people in your community think about mental illness? 4. What do you think are the causes of your relative’s mental illness? |  |
| Service Utilization | 1. Can you please tell me about your experiences from starting of the illness in your relative till now | 1. Where did you visit first for the diagnosis? Why? 2. Where did you visit first for treatment? Why? 3. What factors discourage you from bringing your relative to the hospital for mental health service 4. What factors that encouraged you to seek mental health care from Hospital? 5. What benefits have you derive from bringing your relative to the hospital for mental health services? | *Individual level  *Interpersonal level  *Organizational level  *Community level  *Policy level |
| The burden of care. | Can you please tell me about the stress/difficulties you go through in bringing your relative to the MHS? | 1. How has the mental illness of your relative affected you? Please explain. 2. How do you cope with these challenges or stresses you experience? Please explain. |  |
| Recommendations. | 1. What do you think should be done to address those factors hindering the utilization of mental health services in this municipality? | | |

**KEY INFORMANT INTERVIEW GUIDE FOR CARE PROVIDERS**

I am conducting a study to assess the Family caregivers’ experiences with the utilization of mental health services in selected health facilities in the Bolgatanga municipality. I will be grateful if you could spend a little of your time completing this interview with me. There are no right or wrong answers. Any information provided is private and confidential. This study is only for academic purposes. Your participation in this study is entirely voluntary. Please feel free to answer the questions below. Thank you.

Participant Code……………. Date of interview………………

**Demographics**

**The following questions will be about your demographic characteristics (age, marital status, etc.)**

1. Can you please tell me your age?

2. What is your gender?

3. Where do you residence please?

4. Are you married?

5. What is your highest level of education?

5. How long have you been working in Mental health?

6. What is your job title?

7. Which religion do you belong to?

| **Topics and Issues** | **Main Questions** | **Follow up questions** | **Probe** |
| --- | --- | --- | --- |
| Ice-breaking | Please introduce yourself   1. What are the major Mental services provided by this facility? | Is the delivery of Mental health services by this facility of good quality? If yes/no, please explain. |  |
| Service Utilization | Do people from this community bring their mentally ill relatives for Mental health services? | 1. What factors have you identified as facilitators of the utilization of mental health service in the municipality? 2. What factors have you identified as barriers to the utilization of mental health service in the municipality? | What are the factors affecting mental health service utilization at:  *Individual level.  *Interpersonal level.  *Organizational level.  *Community level.  *Policy level. |
| Recommendations | 1. What are your recommendations for improving mental health service utilization at this municipality? | | |

**KEY INFORMANT INTERVIEW GUIDE FOR MENTAL HEALTH SERVICE ADMINISTRATORS.**

**Demographics**

**The following questions will be about your demographic characteristics (age, marital status, etc.)**

1. Can you please tell me your age?

2. What is your gender?

3. Where do you residence please?

4. Are you married?

5. What is your highest level of education?

5. How long have you been working in Mental health?

6. What is your job title?

7. Which religion do you belong to?

| **Topics and Issues** | **Main Questions** | **Follow up questions** | **Probe** |
| --- | --- | --- | --- |
| Ice-breaking | Please introduce yourself   1. What do you think about the Mental health situation of this municipality? 2. What are the major Mental health services provided by the health facilities in this municipality. | a). What is the quality of mental health service provided in this municipality | Please explain |
| Service Utilization | 1. What are the factors that hinder the utilization of mental health services by people in this municipality. 2. What are the factors that facilitate the utilization of mental health services by people in this municipality | | What are the factors affecting mental health service utilization at:  *Individual level  *Interpersonal level.  *Organizational level (institution)  *Community level  *Policy level |
| Recommendations | 1. What will you recommend for the improvement of mental health service utilization in this municipality? | | |
